# Expressive Suppression of Emotions in Bulimia Nervosa: An Electroencephalography Study

**DOI:** 10.1002/jclp.23761

**Published:** 2024-12-20

**Authors:** Lorena Desdentado, Olga Pollatos

**Affiliations:** ^1^ Clinical and Health Psychology, Institute of Psychology and Education Ulm University Ulm Germany; ^2^ CIBER of Physiopathology of Obesity and Nutrition (CIBEROBN), Instituto de Salud Carlos III Madrid Spain

**Keywords:** bulimia nervosa, eating disorder, EEG, emotion regulation, event‐related potential, suppression

## Abstract

**Objective:**

Previous research has found dysfunctional emotion regulation in bulimia nervosa (BN), including self‐reported greater habitual use of maladaptive strategies such as suppression than in healthy individuals. However, there is no evidence on the performance in the implementation of expressive suppression in BN. The aim of this study was to investigate brain activity (in terms of ERP) and self‐reported ratings associated with expressive suppression of emotions elicited by positive and negative stimuli in women with BN.

**Method:**

Event‐related potentials (ERP) were recorded from 23 female individuals with BN and 26 matched healthy controls. Participants were shown emotional pictures under two conditions: using facial suppression or attentively viewing. High‐density EEG was used to characterize the time course of emotion regulation.

**Results:**

ERP amplitudes varied significantly with valence, with positive (vs. neutral and negative) pictures eliciting larger ERP amplitudes. However, no significant differences in ERP were observed between the groups or conditions. The BN group reported lower self‐efficacy in implementing suppression compared to the control group, the latter with a positive correlation between the perceived self‐efficacy and the change in emotional arousal between conditions.

**Discussion:**

Our findings suggest that individuals with BN might have difficulties in monitoring the emotion regulation process compared to healthy individuals. This suggests that other processes (e.g., metacognitive difficulties, self‐esteem) rather than a failure to implement suppression, might underlie these results. However, further research is needed to validate this interpretation. Implications and directions for future research are discussed.

## 1 Introduction

Eating disorders (ED), most notably anorexia nervosa (AN) and bulimia nervosa (BN), are complex mental disorders that share the psychopathology of over‐evaluating one's own shape and weight, as well as disturbed eating habits or weight control behaviors (Treasure, Duarte, and Schmidt 2020). In particular, individuals with BN typically show frequent episodes of uncontrolled overeating (binge eating), followed by efforts of compensating for the excessive intake (American Psychiatric Association 2013). As pointed out by Treasure, Duarte and Schmidt (2020), there is a need to identify modifiable risk factors for ED, given their increasing prevalence and the serious problems associated with them, such as severe medical complications (Voderholzer et al. 2020) and increased suicide risk (Lipson and Sonneville 2020).

Previous research suggests that substantial difficulties in recognizing emotions and an inability to appropriately manage emotional states are associated with ED (Harrison et al. 2010; Lavender et al. 2015). Fewer studies have focused on BN compared to AN samples (Lavender et al. 2015). Emotion dysregulation is considered so central to the maintenance of EDs that they have been labeled as *emotional disorders* (Bullis et al. 2019). Overall, emotion dysregulation has been associated with more severe ED psychopathology in BN (Lavender et al. 2014), and most conceptual ED models include difficulties in downregulating negative affect as a key aspect. Specifically, it has been suggested that ED‐related behaviors could represent maladaptive attempts to deal with negative affective experiences (Harrison et al. 2010; Heatherton and Baumeister 1991; Lavender et al. 2014; Mallorquí‐Bagué et al. 2018, 2020; Prefit, Cândea, and Szentagotai‐Tătar 2019). Accordingly, individuals with BN show improvements in emotion regulation skills after partial hospitalization treatment with dialectical behavior therapy (Brown et al. 2020).

The extended process model of emotion regulation (Gross 2015b) distinguishes the following stages: (a) *identification*, which determines whether an emotion needs to be regulated; (b) *selection* of a particular emotion regulation strategy; (c) *implementation* of the selected regulatory strategy; and (d) *monitoring* of success (or failure) of these stages. Traditionally, some strategies have been considered *adaptive* across various settings (e.g., acceptance or cognitive reappraisal), whereas others have been regarded as generally *maladaptive* (e.g., rumination or suppression) (Aldao, Nolen‐Hoeksema, and Schweizer 2010). However, recent research suggests that no emotion regulation strategy is universally adaptive or maladaptive across all contexts (Haines et al. 2016; McRae 2016).

*Expressive suppression* is typically described as a *maladaptive* response‐focused strategy (i.e., one that is initiated when the emotion is already underway) that involves hiding, inhibiting, or reducing emotion‐expressive behaviors (e.g., facial expressions, verbal utterances, or gestures) (Goldin et al. 2008). In general, inhibiting negative emotions is considered more demanding and physiologically arousing than not regulating them at all (Danner, Sternheim, and Evers 2014). In this sense, the use of suppression is associated with stress‐related symptoms, such as increased sympathetic activation of the cardiovascular system, greater negative affect, anxiety, depression, and decreased well‐being (Gross 2002; Haga, Kraft, and Corby 2009; Moore, Zoellner, and Mollenholt 2008). Furthermore, individuals who tend to suppress emotions are more likely to engage in secondary emotion regulation strategies, such as emotional eating (Evers, Marijn Stok, and de Ridder 2010). However, the relationship between suppression and mental health symptoms is bidirectional, meaning that suppression is not only a predictor of emotional distress, but may also be a symptom or outcome resulting from psychopathology (Dawel et al. 2021).

According to research using self‐report measures, individuals with BN tend to use expressive suppression of both positive and negative emotions to a greater extent than individuals with AN and healthy controls (Forbush and Watson 2006). Furthermore, a meta‐analytic review by Prefit, Cândea, and Szentagotai‐Tătar (2019) indicated that the self‐reported use of suppression is positively associated with eating pathology. However, it remains unclear whether individuals with BN exhibit specific characteristics in their implementation of expressive suppression beyond the higher self‐reported frequency of using this emotion regulation strategy in daily life. The scarcity of laboratory‐based experimental studies in this field underscores a significant gap in our understanding of the mechanisms underlying emotion regulation processes in BN, as noted by Lavender et al. (2015).

Several imaging studies have investigated the neural basis of emotion regulation, most of which have focused on the downregulation of negative emotions (Braunstein, Gross, and Ochsner 2017; Goldin et al. 2008; Moodie et al. 2020; Ochsner et al. 2004; Phan et al. 2005; Rieck et al. 2024). McRae et al. (2010) summarized that emotion regulation relies upon interactions between prefrontal regions, which can be interpreted as implementing cognitive control, and limbic regions, which can be understood as mediating emotional responses. Sikka et al. (2022) systematically reviewed functional neuroimaging studies on expressive suppression in nonclinical populations, and found that it was associated with increased activation of frontoparietal areas (e.g., dorsolateral and ventrolateral prefrontal cortices) and inferior parietal cortex, and decreased activation in temporo‐occipital regions. Suppression has also been related to other brain regions, such as the anterior insula (which would reflect the role of interoceptive and emotional awareness in emotional suppression) (Giuliani et al. 2011) and the amygdala (Katsumi and Dolcos 2020), but there are mixed results in this regard (Sikka et al. 2022).

While imaging techniques (e.g., functional magnetic resonance imaging [fMRI], positron emission tomography [PET]) allow for identifying brain areas involved in emotional regulation with high spatial accuracy, they lack the fine temporal resolution needed to investigate the time course of such regulation processes. The high temporal resolution (i.e., milliseconds) of electroencephalography (EEG) allows the study of the time course of brain dynamics accompanying emotion regulation, measured through scalp‐recorded electrical activity of the brain. Event‐related brain potentials (ERP) refer to a technique in which the EEG is time‐locked to a specific stimulus, such as the presentation of emotional pictures. Hence, ERP are ideally suited to examine changes in brain functioning during emotion regulation (Weinberg, Ferri, and Hajcak 2013). Two relevant ERP components in the field of emotion regulation are: (1) P300, which is a positive deflection peaking around 300 ms after the onset of the stimulus, and (2) late positive potential (LPP) or slow wave, which can extend for the duration of stimulus presentation (seconds) (Hajcak, MacNamara, and Olvet 2010).

It is well known that the amplitude of the P300 is increased after the presentation of emotional (either positive or negative) stimuli compared to neutral stimuli (Hajcak, MacNamara, and Olvet 2010). When individuals are shown emotional pictures and are asked to downegulate (i.e., suppress) negative affect, the P300 is attenuated compared to a no regulation condition (Reva et al. 2015). However, this is not the case with positive pictures and positive affect. Similarly, previous research has shown that attenuated amplitude of the LPP to negative pictures when implementing an emotion regulation strategy (i.e., reappraisal) is associated with decreases in self‐reported negative emotion (Dickey et al. 2024; Hajcak et al. 2014; Hajcak and Nieuwenhuis 2006; MacNamara, Joyner, and Klawohn 2022), indicating a successful downregulation process at the level of brain activity. Conversely, when a neutral stimulus is pre‐appraised with a negative meaning or a negative stimulus is presented with no regulatory instruction, the LPP is potentiated (Hajcak and Foti 2020; MacNamara, Foti, and Hajcak 2009; Moran, Jendrusina, and Moser 2013). Similarly, Moser et al. (2006, 2009) found reduced ERP amplitudes from 250 ms up to 3500 ms after the onset of the presentation of negative pictures when suppression was instructed compared to viewing (with no regulation instructions). This amplitude obtained with suppression was similar to that observed with neutral stimuli without regulatory instruction (view instruction). Regarding positive emotions, only one study to date has examined the efficacy of expressive suppression to downregulate emotions elicited by positive stimuli (Li et al. 2020). This study found that expressive suppression (vs. free viewing) attenuated both early (500–700 ms) and late (700–1500 ms) LPP responses to positive pictures of both high and low intensity. However, to the best of our knowledge, no studies have examined the brain dynamics associated with expressive suppression of both positive and negative affect in BN.

The aim of this study was to examine brain activity (in terms of ERP) and self‐reported ratings accompanying expressive suppression of emotions elicited by positive and negative stimuli in individuals with BN compared to healthy individuals. We hypothesized that individuals with BN would show less attenuation of both P300 and LPP when using expressive suppression (vs. free viewing) when exposed to positive and negative pictures compared to healthy controls. No differences between groups were expected for neutral stimuli. We also expected greater downregulation of emotional arousal with the use of suppression (vs. viewing) in individuals without (vs. with) BN. This hypothesis is based on previous research indicating that individuals with higher levels of emotional eating exhibit impaired inhibitory control, especially when attempting to suppress negative emotions (Wolz, Biehl, and Svaldi 2021). Emotional eating is known to play a pivotal role in BN (Meule et al. 2021; Ricca et al. 2012). In addition, inhibitory control deficits have been observed in individuals with BN, even when confronted with general (non‐disease‐salient) stimuli (Wu et al. 2013). Given this presumed inhibitory control deficit, we expected individuals with BN to exhibit less downregulation of emotional arousal when using suppression compared to controls.

## 2 Methods

### 2.1 Participants

A total of 23 female individuals (*M*_age_ = 24.3, SD = 7.2, range: 15–44 years old) with current BN were recruited from local consultation units for EDs (i.e., ANAD e.V., Pathways, Caritas Ambulance, Cinderella e.V., TCE Therapy Center) and paid for participation. All of them met the criteria for BN as assessed by the Structural Clinical Interview for DSM‐III‐R Axis I Disorders (SCID) (Spitzer 1992). ICD‐10/DSM‐IV criteria of BN were also considered. Individuals receiving psychotropic medication, including antidepressants and pain relievers, were excluded from the study. On average, the course of the disorder lasted 7.3 years (SD = 6.4 years), with the onset of the disorder at a mean age of 16.7 years (SD = 3.1 years). The mean body mass index (BMI) was 20.8 kg/m^2^ (SD = 3.4 kg/m^2^).

A total of 26 healthy female individuals of similar age (*M*_age_ = 25.1, SD = 3.2, range: 20–34 years old) were recruited from Ulm University for the control group, who received either course credit or money for participation. All of them reported no past or current ED and no current use of medication. Their mean BMI was 22.1 kg/m^2^ (SD = 2.5 kg/m^2^).

### 2.2 Measures

Several self‐report questionnaires were administered to characterize the sample, which are described below.

The *Restraint Scale* (RS) (Herman and Polivy 1980) is a widely used self‐report measure for assessing dietary restraint and weight fluctuation. The RS includes 10 items rated from 0 (*never*) to 4 (*always*). Higher total scores indicate a greater degree of restrained eating behavior (Dinkel et al. 2005). The internal consistency found in this study was adequate (*α* = 0.93; *ω* = 0.95).

The “Interoceptive Awareness” subscale of the *Eating Disorder Inventory* (EDI‐IA) includes 10 items rated on a 4‐point Likert scale, ranging from 0 (*never*) to 3 (*always*) and measures a lack of confidence in identifying emotions and sensations of satiety and hunger (Garner, Olmstead, and Polivy 1983). Higher scores reflect lower interoceptive sensibility. The internal consistency of this measure in this study was adequate (*α* = 0.93; *ω* = 0.96).

The *Toronto Alexithymia Scale‐20* (TAS‐20) (Bagby, Parker, and Taylor 1994) is a widely used questionnaire for the assessment of alexithymia. It includes 20 items rated on a 5‐point Likert scale (1 = *strongly disagree*; 5 = *strongly agree*) and comprises the following dimensions: difficulties describing feelings (DDF), difficulties identifying feelings (DIF), and externally oriented thinking (EOT). A total score can also be computed, with higher scores indicating more severe alexithymia. In this study, the internal consistency of the TAS‐20 was adequate except for the EOT dimension, which is consistent with previous studies (Schroeders, Kubera, and Gnambs 2022) (DDF: *α* = 0.75; *ω* = 0.82; DIF: *α* = 0.91; *ω* = 0.94; EOT; *α* = 0.53; *ω* = 0.64; total score: *α* = 0.86; *ω* = 0.88).

The *Spielberger State Trait Anxiety Index* (STAI) (Spielberg, Gorsuch, and Lushene 1970) was used to measure anxiety. It includes 20 items for state anxiety (STAI‐S) and 20 items for trait anxiety (STAI‐T), all rated from 0 (*not at all*) to 3 (*very much*). Higher scores reflect more severe anxiety. The internal consistency of this instrument found in this study was appropriate (STAI‐S: *α* = 0.87; *ω* = 0.91; STAI‐T: *α* = 0.94; *ω* = 0.96).

The *Beck Depression Inventory‐II* (BDI‐II) (Beck, Steer, and Brown 1996) was administered to assess depressive symptomatology. It includes 21 items rated from 0 to 3. Total scores range from 0 to 63, with higher scores indicating greater severity. The internal consistency found in this study was adequate (*α* = 0.92; *ω* = 0.95).

In addition, the SCID was also administered, as reported in the Section 2.1. The positive, neutral, and negative pictures presented (as described in the Section 2.3) were also rated on a 10‐point Likert scale ranging from 1 to 9 for valence and arousal at the end of the experiment. Higher scores reflect a more positive valence and greater arousal, respectively.

### 2.3 Stimuli and Procedures

The current study was conducted in accordance with the Declaration of Helsinki and its later amendments and was approved by an institutional review board. Written informed consent was obtained from all participants.

First, height was measured with a tape measure placed on the wall and weight was measured with a weighing machine. Next, participants were asked to complete a series of self‐report questionnaires (see Section 2.2), including sociodemographic and clinical information. Afterwards, the formal experiment was administered.

A total of 180 pictures (60 neutral, 60 positive, 60 negative) from the International Affective Picture System (IAPS) (Lang, Bradley, and Cuthbert 1995) were used as emotional stimuli to evoke neutral, positive, or negative emotional states, based on the valence scores reported in the manual. Negative pictures included images such as frightening animals and mutilated human bodies; positive pictures included images such as cute animals; and neutral pictures depicted daily necessities such as tableware and books.

The experimental protocol used a block design, presenting two conditions (“view” and “suppression”) in a counterbalanced order. Within each condition, blocks with different emotional stimuli (neutral, positive, and negative) were also presented in a counterbalanced order. In the “view” condition, participants were asked to maintain their natural emotional response without altering it. In the “suppression” condition, participants were instructed to inhibit their expressive behaviors to hide their emotional state (i.e., maintain a poker face). Additionally, they were told that facial information would be recorded and rated afterwards to ensure that they attempted to keep a poker face. Thus, the unique blocks included in the study were as follows: view‐neutral, view‐negative, view‐positive, suppression‐neutral, suppression‐positive, and suppression‐negative. Each block involved the presentation of a total of 30 pictures, either neutral, positive, or negative.

Participants were seated in a comfortable chair in a dimly lit, sound‐attenuated chamber, with a 19‐inch computer screen placed ~140 cm away, centered in their field of vision. Each trial began with a fixation cross for 500 ms, followed by a variable interstimulus interval of 250–500 ms, after which an IAPS picture was displayed for 5 s. This duration was based on previous studies using similar procedures the field of emotion regulation (Baur et al. 2015; Füstös et al. 2013; Moran, Jendrusina, and Moser 2013; Sarlo et al. 2013) and aligns with evidence indicating that the LPP in response to emotional stimuli lasts several seconds from the onset of the presentation (Hajcak, MacNamara, and Olvet 2010). A variable time interval of 1.5–3 s was applied before the next trial.

**Figure 1:**
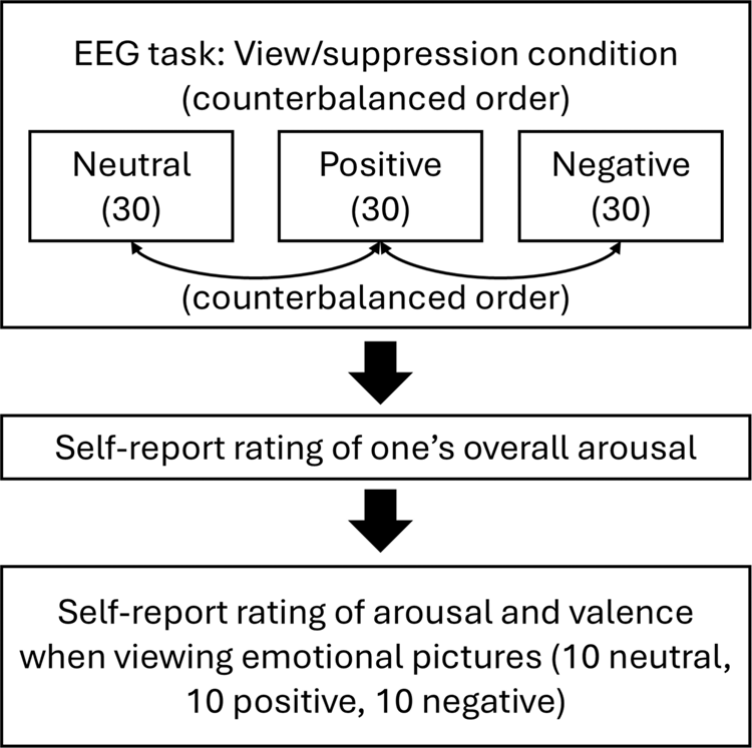
Overview of the study protocol.

Overall arousal was rated after each condition (“view” and “suppression”). Following the “suppression” condition, participants rated how well they were able to suppress their facial expressions on a 9‐point rating scale, with higher scores indicating greater self‐reported regulatory skills. Finally, after the experimental protocol, participants provided valence and arousal ratings for another 30 pictures from the IAPS (i.e., 10 neutral, 10 positive, and 10 negative) on a 9‐point scale using the Self‐Assessment Manikin (SAM) (Bradley and Lang 1994). They rated how pleasant and aroused they felt while viewing each picture, with scores ranging from 1 (*very unpleasant* or *low aroused*) to 9 (*very pleasant* or *high aroused*). This task allowed us to capture emotional processing at the self‐report level in both the BN and control groups. The images used in this self‐report task were comparable in terms of valence and arousal to those used in the EEG task. An overview of the study protocol is shown in Figure 1.

Participants were instructed to avoid looking away (unless necessary), making exploratory eye movements, blinking, and becoming distracted while viewing the pictures in both conditions. Before the experimental testing, a short training block was conducted using five pictures that were not included in the analysis.

### 2.4 Data Recording and Analyses

#### 2.4.1 EEG Recording

EEG data were collected from 64 channels following the 5% system (Oostenveld and Praamstra 2001), using a DC amplifier (bandpass: 0.01–100 Hz; SYNAMPS, Neuroscan) and digitized at a sampling rate of 1000 Hz. The electrode placements were guided by an electrode cap (Falk Minow Services). Cz served as the reference electrode, while the ground electrode was positioned on the left cheek. Offline re‐referencing was performed to linked mastoids. Eye movements, both horizontal and vertical, were monitored using electrodes positioned at the outer corners of each eye (EOG_H_) and above and below the left eye (EOG_V_). Nonpolarizable Ag–AgCl electrodes were used, and the electrode resistance was maintained below 8 KΩ.

#### 2.4.2 EEG Data Reduction and Analysis

The EEG data were inspected for electrooculography (EOG), muscle activity, and other electrophysiological artifacts. Blink artifacts were corrected using the algorithm by Gratton, Coles and Donchin (1983), as implemented in the Vision Analyzer software (Brain Products). The EEG signals were filtered with a bandpass of 0.01–30 Hz and averaged offline. Epochs were extracted with a 1000 ms window starting at the onset of the picture presentation, relative to a 200 ms prestimulus baseline.

After segmentation and preprocessing, epochs were averaged within each block and each of the 12 regions of interest, which were formed by crossing the hemisphere (right/left) with the horizontal plane (anterior, medial, posterior), and the vertical plane (inferior, superior) (Keil et al. 2002; Pollatos, Gramann, and Schandry 2007). The resulting ERP waveform for each region reflects the average response across all repetitions within each block, representing the neural response typical for each condition. Finally, we computed the average for the frontal (i.e., anterior inferior right, anterior inferior left, anterior superior right, anterior superior left) and central (i.e., medial inferior right, medial inferior left, medial superior right, medial superior left) areas for both P300 and LPP. As the activity in the posterior areas is not relevant for emotion regulation, they were omitted.

#### 2.4.3 Statistical Analyses

Data management, internal consistency, plots, and descriptive analyses were performed in R 4.3.1, whereas inferential statistics were performed in SPSS 29. Mixed ANOVAs and *t*‐tests were used to analyze self‐reported and EEG data as specified in the Section 3. Where the assumptions for the two‐sample *t*‐tests were not met (see Table S1), the Welch‐test was reported, as it is a powerful and robust alternative that performs well in most scenarios (Rasch, Kubinger, and Moder 2011). Following the recommendations by Merino and San Martín Castellanos (2010) for mixed ANOVAs, when multisample sphericity could not be assumed based on Box's *M* test and Mauchly's test of sphericity (see Table S2), the statistics Pillai's trace, Wilks' lambda, Hotelling's trace, and Roy's largest root were considered for within‐subject effects. However, it should be noted that the results did not substantially change when considering *F* statistics with or without the assumption. For significant main and interaction effects, post hoc tests were performed with Bonferroni correction.

Additionally, correlation and moderation analyses were performed to test whether the relationship between the delta arousal (differential arousal reported after the “view” and “suppression” conditions) and perceived success in implementing suppression differed between individuals with and without BN. To do so, “model 1” of the macro PROCESS (Hayes 2013) was used, where *Y* was the perceived success in suppression, *X* was the delta arousal, and *W* was the group (BN, control). Scores on *X* were mean‐centered for interpretability. Regression coefficients are reported in unstandardized form. The control group was coded as “1” and the BN group was coded as “2.”

## 3 Results

### 3.1 Characteristics of the Sample

Table 1 presents the sociodemographic and questionnaire data for individuals with and without BN. *T*‐tests indicated that individuals with BN reported significantly more disturbed eating attitudes and behaviors than controls, as measured by the RS. In addition, participants with BN showed significantly lower scores on interoceptive sensibility (EDI‐IA) than those in the control group. Participants with (vs. without) BN also reported significantly higher scores on two out of three TAS‐20 dimensions (i.e., DDF and DIF), as well as on the total score, indicating higher levels of alexithymia, whereas a nonsignificant trend was observed for the EOT dimension. Finally, individuals with BN also showed higher levels of both state and trait anxiety (STAI) and depressive symptomatology (BDI‐II) compared to the control group. There were no significant differences in age or BMI between the groups. Table S3 shows the correlations between the self‐report and the electrocortical variables. Overall, higher BMI and poorer scores on self‐report questionnaires are associated with lower ERP amplitudes.

**Table 1:** Sociodemographic and questionnaire data of BN and control groups.

	Mean (SD)		*t‐*test		Effect size	
Variable	Control group	BN group	*t*	*p* value	*d*	95% CI
Age (years)	24.92 (3.15)	24.043 (7.18)	0.54	0.593	0.16	[−0.39, 1.01]
BMI	21.79 (2.51)	20.88 (3.4)	1.05	0.297	0.3	[−0.27, 0.97]
RS	**9.2 (5.68)**	**22.91 (6.05)**	**−8.1**	**< 0.001**	**−2.34**	**[−3.28, −1.73]**
EDI‐IA	**48.75 (5.3)**	**30.83 (9.19)**	**8.14**	**< 0.001**	**2.39**	**[1.86, 3.42]**
TAS‐20 (DDF)	**9.56 (3.16)**	**15.17 (3.75)**	**−5.62**	**< 0.001**	**−1.62**	**[−2.78, −0.97]**
TAS‐20 (DIF)	**11.92 (3.51)**	**21.3 (5.96)**	**−6.57**	**< 0.001**	**−1.92**	**[−3.03, −1.3]**
TAS‐20 (EOT)	15.56 (3.31)	17.65 (4.26)	−1.91	0.062	−0.55	[−1.27, 0.02]
TAS‐20 (Total)	**37.04 (7.37)**	**54.13 (8.9)**	**−7.27**	**< 0.001**	**−2.1**	**[−3.45, −1.35]**
STAI‐S	**35.52 (5.08)**	**42.48 (9.04)**	**−3.32**	**0.002**	**−0.96**	**[−1.59, −0.47]**
STAI‐T	**39.28 (9.92)**	**54.91 (11.41)**	**−5.08**	**< 0.001**	**−1.47**	**[−2.49, −0.81]**
BDI‐II	**4.42 (3.98)**	**18.41 (9.97)**	**−6.15**	**< 0.001**	**−1.84**	**[−2.78, −1.2]**

*Note:* Statistically significant differences between groups are shown in bold. Homogeneity of variances was not assumed for age, EDI‐IA, TAS‐20 (DIF), and BDI‐II.

Abbreviations: BDI‐II, Beck Depression Inventory; BMI, body mass index; BN, bulimia nervosa; CI, confidence interval; DDF, difficulties describing feelings; DIF, difficulties identifying feelings; EDI‐IA, Interoceptive Awareness subscale of the Eating Disorder Inventory; EOT, externally oriented thinking; RS, Restrained Scale; SD, standard deviation; STAI, Spielberger State Trait Anxiety Index; TAS‐20, Toronto Alexithymia Scale‐20.

### 3.2 Results of Self‐Report Ratings

**Figure 2:**
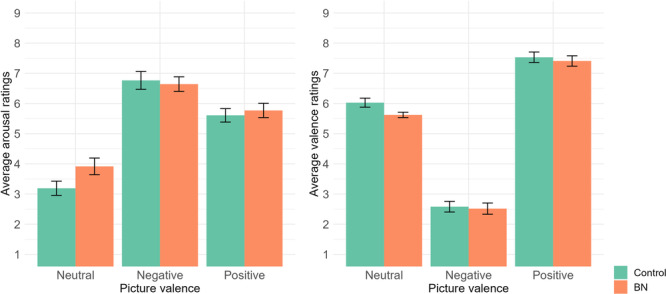
Arousal and valence ratings for neutral, negative, and positive pictures in individuals with and without BN.

Figure 2 shows the arousal and valence ratings for the IAPS pictures (neutral, negative, and positive) in participants with and without BN. We performed two‐way ANOVAs with a between‐subjects factor for group (BN, control) and a within‐subjects factor for picture valence (neutral, negative, and positive) for both arousal and valence ratings.

Results indicated a main effect of picture valence on *arousal* ratings, *F* (2, 45) = 104.92, *p* < 0.001, *η*_p_^2^ = 0.823. Negative pictures were rated with the highest arousal, whereas neutral pictures were rated with the lowest arousal. Positive pictures had significantly lower arousal ratings than negative pictures and significantly higher ratings than neutral pictures (*p* < 0.001 in all post hoc comparisons). However, no significant effects were found for group, *F* (1, 46) = 0.781, *p* = 0.382, *η*_p_^2^ = 0.017, or for the interaction, *F* (2, 45) = 2.76, *p* = 0.078, *η*_p_^2^ = 0.057.

Similarly, as regards *valence* ratings, only picture valence showed significant effects, *F* (2, 45) = 226.95, *p* < 0.001, *η*_p_^2^ = 0.910. Negative pictures were rated with the lowest valence, whereas positive pictures received the highest valence. Negative pictures had significantly lower valence ratings than neutral and positive pictures (*p* < 0.001 in all post hoc comparisons). However, no significant effects were found for group, *F* (1, 46) = 3.375, *p* = 0.073, or for the interaction, *F* (2, 45) = 2.044, *p* = 0.141.

**Figure 3:**
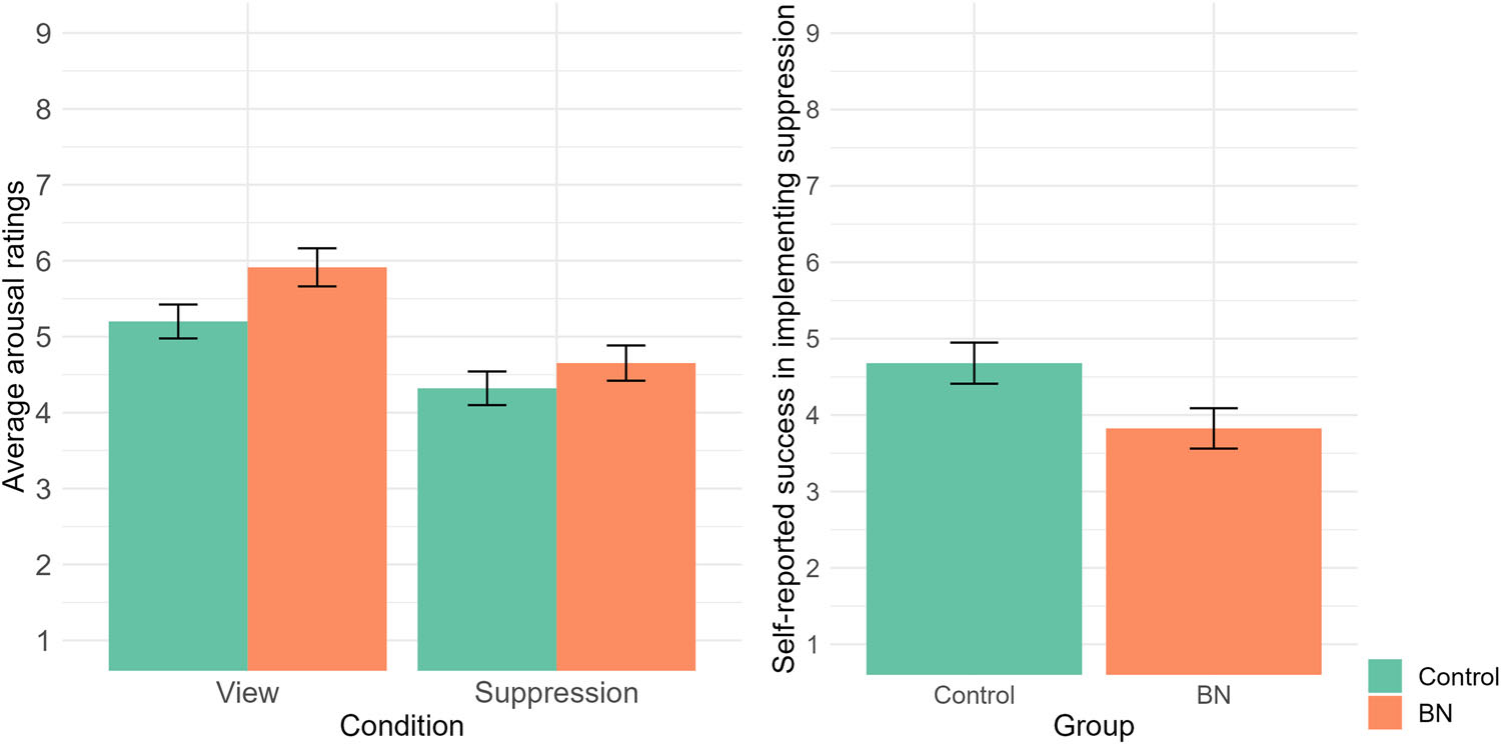
Arousal ratings after the view and the suppression conditions in individuals with and without BN and self‐reported success in implementing suppression.

Regarding the emotional arousal perceived after the experimental conditions (Figure 3), a two‐way ANOVA with a between‐subjects factor for group (BN, control) and a within‐subjects factor for condition (suppression, view) reveled that only condition had a significant main effect, *F* (1, 46) = 41.35, *p* < 0.001, *η*_p_^2^ = 0.473. Specifically, participants reported lower emotional arousal in the “suppression” than in the “view” condition (*p* < 0.001). However, no significant effects were found for group, *F* (1, 46) = 3.423, *p* = 0.071, *η*_p_^2^ = 0.069, or for the interaction, *F* (1, 46) = 1.309, *p* = 0.259, *η*_p_^2^ = 0.028.

Regarding the self‐reported ability to successfully implement suppression (Figure 3), a *t*‐test showed that individuals with BN reported lower self‐efficacy than healthy controls, *t*(46) = 2.259, *p* = 0.029, *d* = 1.31, 95% CI = [0.07, 1.23].

**Figure 4:**
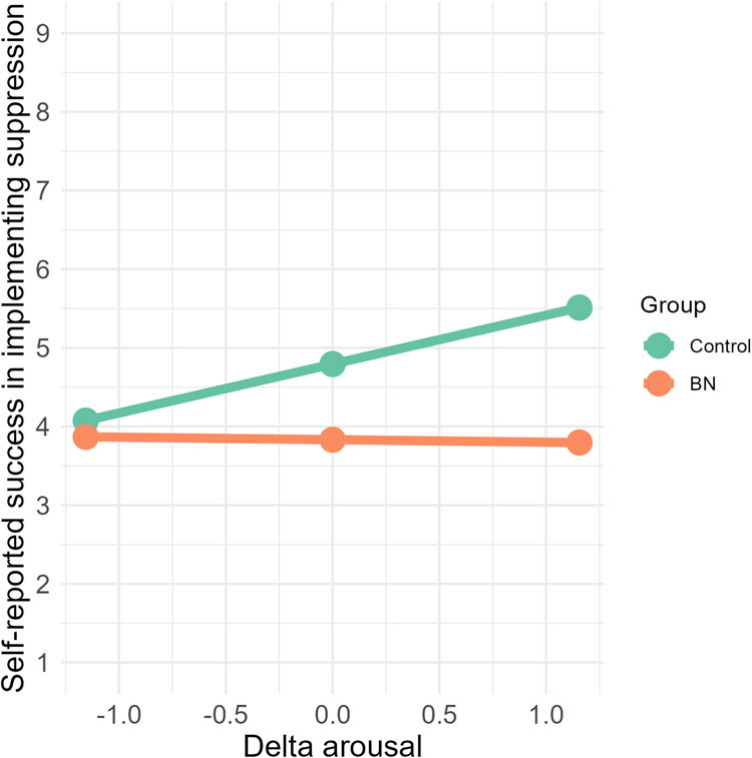
Simple slopes graph of the relationship between delta arousal and perceived success in suppressing emotions for individuals with and without BN.

It should be noted that perceived success in suppressing was positively correlated with delta arousal in the control group (*r* = 0.52, *p* = 0.007), whereas this relationship was not statistically significant in the BN group (*r* = −0.03, *p* = 0.895). A moderation analysis supported that the relationship between self‐reported success and delta arousal significantly differed between groups (see Table 2). The overall model was statistically significant, *F* (3, 44) = 4.52, *p* = 0.008, *R* ^2^ = 0.24. Analyses of simple slopes showed a positive relationship between delta arousal and perceived success in suppressing emotions for individuals with BN (*b* = 0.62, SE = 0.22, *p* = 0.008, 95% CI = [0.17, 1.07]), whereas this relationship was not statically significant for individuals without BN (*b* = −0.03, SE = 0.22, *p* = 0.889, 95% CI = [−0.48, 0.42]) (Figure 4), consistent with the Pearson's correlations observed.

**Table 2:** Results of the moderation model for perceived success in suppressing emotions.

	*b* [95% CI]	SE	*t*	*p* value
Intercept	**5.75 [4.61, 6.89]**	**0.56**	**10.21**	**< 0.001**
Delta arousal	**1.27 [0.27, 2.28]**	**0.50**	**2.56**	**0.014**
Group	**−0.96 [−1.69, −0.23]**	**0.36**	**−2.66**	**0.011**
Delta arousal × Group	**−0.65 [−1.29, −0.02]**	**0.32**	**−2.07**	**0.044**

*Note:* Statistically significant differences between groups are shown in bold.

Abbreviations: CI, confidence interval; SE, standard error.

### 3.3 ERP Results

**Figure 5:**
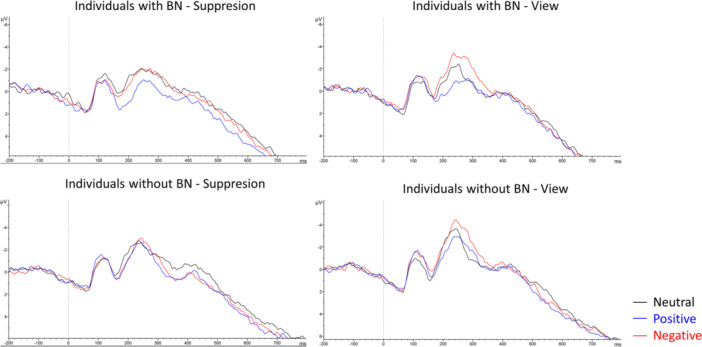
Right anterior inferior ERP in individuals with and without BN across conditions (view and suppression) and emotional stimuli (neutral, positive, and negative).

Figure 5 shows the ERP exemplarily in the right anterior inferior region for suppression and view conditions for neutral, negative, and positive stimuli in individuals with and without BN. To examine the neural regulation of expressive suppression (vs. free viewing) between individuals with and without BN, we performed three‐way mixed ANOVAs on both P300 and LPP magnitudes. These analyses included group (BN, control) as a between‐subjects factor, with condition (suppression, view) and picture valence (negative, positive, and neutral) as within‐subjects factors (see Table 3).

**Table 3:** ANOVA results for P300 and LPP in frontal and central regions.

	*F*	*df*	*p* value	*η*_p_^2^
*P300 frontal*				
Group	0.044	1, 47	0.835	0.001
Valence	**5.88**	**2, 46**	**0.005**	**0.203**
Valence × group	1.37	2, 46	0.264	0.056
Condition	0.10	1, 47	0.749	0.002
Condition × group	0.07	1, 47	0.799	0.001
Valence × condition × group	0.32	2, 46	0.726	0.014
*P300 central*				
Group	0.09	1, 47	0.772	0.002
Valence	**15.68**	**2, 46**	**< 0.001**	**0.250**
Valence × group	0.01	2, 46	0.988	0.000
Condition	1.84	1, 47	0.181	0.038
Condition × group	1.12	1, 47	0.296	0.023
Valence × condition × group	0.13	2, 46	0.880	0.003
*LPP frontal*				
Group	0.09	1, 47	0.762	0.002
Valence	**4.16**	**2, 46**	**0.019**	**0.081**
Valence × group	0.65	2, 46	0.527	0.014
Condition	2.78	1, 47	0.102	0.056
Condition × group	0.02	1, 47	0.893	0.000
Valence × condition × group	0.28	2, 94	0.758	0.006
*LPP central*				
Group	0.646	1, 47	0.426	0.014
Valence	**7.37**	**2, 46**	**0.001**	**0.136**
Valence × group	0.315	2, 46	0.730	0.007
Condition	1.32	1, 47	0.257	0.027
Condition × group	1.38	1, 47	0.246	0.033
Valence × condition × group	0.549	2, 46	0.579	0.023

*Note:* Statistically significant effects are shown in bold.

For the P300 in frontal regions, results revealed a significant main effect of picture valence. Post hoc comparisons showed that positive pictures elicited greater ERP amplitude than neutral pictures (*p* = 0.004), while no other pairwise comparisons reached statistical significance. No significant effects were found for group, condition, or interactions.

For the P300 in central regions, a similar pattern was observed, with picture valence as the only significant effect (see Table 3). Post hoc comparisons showed that positive pictures elicited greater ERP amplitude than both negative (*p* = 0.033) and neutral pictures (*p* < 0.001). Moreover, negative pictures elicited greater ERP amplitude than neutral pictures (*p* = 0.004).

For the LPP in frontal regions, picture valence was the only significant effect observed. Pairwise comparisons showed that positive pictures elicited greater ERP amplitude than neutral pictures (*p* = 0.045), while no other pairwise comparisons reached statistical significance. No significant effects were found for group, condition, or interactions.

For the LPP in central regions, results indicated a significant main effect of picture valence. Pairwise comparisons showed that positive pictures elicited greater ERP amplitude than negative pictures (*p* = 0.006), which in turn had greater ERP amplitude than neutral pictures (*p* = 0.013). However, there were no significant differences between positive and negative pictures (*p* = 1.00). Again, no significant effects were found for group, condition, or interactions.

Finally, it is worth noting that the main results reported above did not change substantially when BMI is included as a covariate.

## 4 Discussion

Although difficulties in emotion regulation, particularly the increased use of expressive suppression as a regulatory strategy, have attracted substantial interest in ED research, little is known about the performance of this strategy and its underlying neural mechanisms. The current study examined suppression performance at both the self‐report and electrophysiological levels in response to emotional stimuli in individuals with and without BN.

Overall, positive, neutral, and negative pictures were rated as expected in terms of valence and arousal in both groups, indicating no significant differences in emotional processing between individuals with BN and controls. This finding is consistent with previous research showing that individuals with BN (as well as AN) do not rate the valence or arousal of standard emotional pictures (i.e., IAPS) significantly different from healthy participants (Blechert et al. 2011). However, this does not extend to the emotional processing of other types of stimuli considered disorder‐relevant, such as food cues (Delgado‐Rodríguez et al. 2019) or overweight body shape (Spangler and Allen 2012), where individuals with BN have shown dysregulated emotional processing compared to healthy participants.

When participants (both with and without BN) were exposed to emotional stimuli and instructed to suppress their emotional response, their arousal scores decreased compared to when they simply viewed the emotional stimuli without using any emotion regulation strategy. However, contrary to our hypothesis, no significant differences were found between the two groups. In other words, suppression (vs. viewing) was associated with lower overall arousal ratings. Although there were no differences in arousal ratings between the BN and the control groups, participants with BN reported lower success in suppressing emotions compared to those in the control group. Additionally, a positive relationship between perceived success in implementing suppression and actual reduction in arousal (measured by the delta arousal between the suppression and view conditions) was observed only in healthy individuals. In contrast, perceived success and performance in suppressing emotions were not significantly related in individuals with BN.

In summary, these findings suggest that while suppression helped reduce overall arousal ratings in both groups, only the control group showed an association between this reduction and greater self‐efficacy in using suppression. People with BN exhibited low self‐efficacy in using suppression, regardless of how much they reduced their arousal in the suppression (vs. view) condition. In contrast, in the control group, those who significantly reduced their arousal in the suppression condition demonstrated higher self‐efficacy in implementing suppression (and vice versa). This result can be interpreted as an indicator of a poorer metacognition of emotion regulation (i.e., reduced awareness of one's own performance in emotion regulation) in the BN group compared to the control group. Monitoring regulatory success has been highlighted as a crucial stage of the emotion regulation process, as it informs whether to continue or stop using a certain emotion regulation strategy (McRae and Gross 2020). This deficit might be a potential source of dysfunctional patterns of emotion regulation, such as emotion overregulation, where emotion regulation processes might remain active even when the need for regulation has passed (Gross 2015a). Furthermore, monitoring enhances the ability to flexibly switch between strategies (e.g., adopting a different strategy when the current one is ineffective). This is particularly true for expressive suppression, as its adaptiveness can vary significantly throughout the day. For instance, there are situations where inhibiting the expression of positive emotions is maladaptive (e.g., when a friend shares good news) and others where inhibiting the expression of negative emotions is adaptive (e.g., when you are upset after a fight with your partner and encounter a co‐worker in the hallway). However, it should be noted that our study was not primarily designed to address this aspect, and research on emotion regulation monitoring is still in its infancy (Roelofs, Bramson, and Toni 2023).

Still, our findings are consistent with emerging evidence on the role of metacognitions in ED. For instance, a meta‐analysis found dysfunctional metacognitions across various psychiatric disorders, including ED, such as reduced confidence in one's cognitive abilities and an increased belief in the need to control thoughts (Sun, Zhu, and So 2017). Along these lines, Lysaker et al. (2023) found that metacognition—defined as the ability to form an integrated sense of self and others, as measured by the Metacognition Assessment Scale‐Abbreviated (Lysaker et al. 2020)—was negatively related to aspects of general psychopathology in individuals with BN. However, the link between metacognitive skills and emotion regulation in BN has not yet been explored. Future studies should investigate whether a metacognitive failure to monitor the success in changing the trajectory of emotions (i.e., the target of emotion regulation processes) underlies the greater use of maladaptive regulatory strategies (such as suppression) in people with BN (vs. healthy individuals) found in previous studies (e.g., Forbush and Watson 2006).

Another possible explanation (not mutually exclusive) for our results may involve lower levels of self‐esteem in the BN group compared to the control group. In other words, individuals with BN might have evaluated themselves more negatively, regardless of their performance. Although we did not measure self‐esteem in this study, previous research has identified deficits in self‐esteem as a core symptom of ED (e.g., Daley et al. 2008; Shea and Pritchard 2007). Furthermore, meta‐analytic evidence has shown that psychotherapy for BN leads to improvements in self‐esteem (Linardon, Kothe, and Fuller‐Tyszkiewicz 2019). Future research should elucidate the interplay between self‐esteem and emotion regulation in BN.

At the electrophysiological level, and contrary to our expectations, the valence of the emotional pictures was the only factor that had significant effects on the ERP amplitudes in both P300 and LPP (including both frontal and central regions), with positive pictures being associated with larger overall amplitudes, as observed in previous studies (Bernat et al. 2011; Myruski et al. 2019). Unlike overall arousal ratings, ERP amplitudes were not significantly influenced by the instruction (suppression or view). Although this lack of a significant effect contrasts with the well‐documented increase in sympathetic activation associated with expressive suppression (Gross 1998, 2002), previous ERP studies on the effects of expressive suppression have yielded mixed results. For instance, Pan, Wang, and Li (2019) found that suppression was associated with reduced LPP amplitudes in individuals with high (vs. low) trait anxiety. They also reported a positive correlation between trait anxiety and the self‐reported habitual use of suppression, indicating success in regulating emotions using this maladaptive strategy, which is common in their repertoire. This contrasts with our finding of no differences between individuals with BN, who typically exhibit greater habitual use of suppression, and controls. Other studies have also shown smaller LPP and P300 amplitudes in suppression (vs. viewing/maintaining) conditions (Lusk et al. 2017; MacNamara, Joyner, and Klawohn 2022; Moser et al. 2006, 2009; Zhu et al. 2019). Conversely, some studies found no significant effects of suppression on LPP (Lusk et al. 2017) or even an opposite tendency, such as increased LPP (Bernat et al. 2011; Li et al. 2020). These inconsistent findings suggest that the neural correlates of the use of suppression may be moderated by other variables, such as gender. In this sense, our results would be consistent with the study by Myruski et al. (2019), which found that male participants showed significantly greater reductions in LPP associated with suppression (vs. ‐maintain‐control condition) of emotions elicited by positive stimuli, whereas no significant differences were observed in female participants.

Our self‐report data findings differ from our ERP findings. Self‐reported arousal ratings were the only indicator of successful emotion downregulation using suppression (vs. passive viewing) in both individuals with and without BN. The discrepancy between self‐reported and physiological effects of suppression has been noted in previous studies. For instance, Goldin et al. (2008) found that suppression was associated with changes in brain regions involved in inhibitory control (e.g., prefrontal cortex) only during a later period (10.5–15 s). Given the shorter time window covered by ERPs, this neurophysiological marker may not reliably capture the effects of suppression, potentially explaining the mixed results in previous literature. Additionally, discrepancies between self‐reported and electrocortical effects of suppression have been noted in the opposite direction. For example, Takehara, Ishihara, and Iwaki (2020) observed greater right frontal alpha activity when participants suppressed their facial expressions compared to facilitating them, even though no differences were found in their perception of positive vs. negative image stimuli. Future studies should further investigate the mechanisms underlying the divergent effects of the use of emotion regulation strategies from a multimodal perspective to better understand dysfunctional emotion regulation patterns in clinical conditions such as ED.

Some theoretical and clinical implications can be tentatively derived from this study. First, our findings highlight the importance of considering other cognitive processes (e.g., metacognition) and emotional factors (e.g., self‐esteem) to better understand emotion regulation in BN. Interventions aimed at improving emotion regulation in individuals with BN should address these underlying mechanisms of emotion dysregulation. Furthermore, both theoretical and therapeutic approaches should distinguish between the habitual use of emotion regulation strategies and their actual effectiveness. Future research should also explore various emotional regulation outcomes, both self‐reported and physiological, as these may not always align. Investigating this discrepancy should be a focus of future studies.

This study is not exempt from limitations that should be mentioned. First, the characteristics of the sample are limited and cannot be generalized to other populations, such as individuals with a different ED such as AN, or male or nonbinary individuals. Second, our study used emotional pictures as stimuli to evoke positive and negative emotions. Although the standardized IAPS set is widely used in the field of emotion regulation, particularly in studies measuring ERP components (e.g., Bernat et al. 2011; Li et al. 2020), it is limited in terms of ecological validity (MacNamara, Joyner, and Klawohn 2022). Future research using ERPs to examine emotion regulation should use novel methods that better represent real‐life emotion regulation, such as idiographic approaches (e.g., autobiographical emotion regulation task) (Speed et al. 2020). Third, the sample size in this study was relatively small, which may limit the statistical power to detect significant effects. However, it is worth noting that our sample size is comparable to, or even larger than, those used in much of the research in this field (e.g., Blechert et al. 2011; Li et al. 2020; Moser et al. 2009; Sikka et al. 2022). Finally, it is important to note that this study exclusively examined time‐domain components of brain activity. Future research incorporating spectral analysis of the EEG signal could yield additional insights into regulatory mechanisms not captured by ERP components such as P300 and LPP.

In conclusion, the efficacy of expressive suppression was assessed at both self‐report and electrocortical levels in individuals with and without BN. Our findings indicate that expressive suppression effectively modulated self‐reported emotional arousal in both groups, but this effect was not observed at the neurophysiological level. Notably, the success of suppression—measured by the extent to which overall arousal ratings were modulated in the suppression vs. control condition—was associated with self‐efficacy in using this strategy for individuals without BN, but not for those with BN. These results highlight a discrepancy between perceived and actual performance in emotion regulation among individuals with BN.

## Ethics Statement

This study was approved by the Ethics Committee of Ulm University and was conducted in according with the Declaration of Helsinki and its later amendments. All participants signed the informed consent before participating in the study.

## Conflicts of Interest

The authors declare no conflicts of interest.

## Supporting information

Supporting information.

## Data Availability

The data sets and the analytic codes used in this study are publicly available at: https://osf.io/2k63v/.
