# Supplementary material for: Expressive Suppression of Emotions in Bulimia Nervosa: An Electroencephalography Study
Source: J Clin Psychol. 2024 Dec 20;81(3):158–70. doi: 10.1002/jclp.23761 (PMC11802486; doi:10.1002/jclp.23761)
Supplement: Supplementary file 1 — Supporting information. [file JCLP-81-158-s001.docx]

**Table S1.** Normality and homogeneity of variance tests for variables tested for comparison of two independent samples (t-test or Welch-test)

| **Variable** | **Normality tests** | | | | **Homogeneity of variance tests** | |
| --- | --- | --- | --- | --- | --- | --- |
|  | **BN group** | | **Control group** | |  |  |
|  | **Shapiro test** | ***p*** | **Shapiro test** | ***p*** | **Levene’s test** | ***p*** |
| Age | .86 | .004 | .92 | .062 | **6.03** | **.018** |
| BMI | .94 | .192 | .95 | .266 | 1.23 | .274 |
| RS | .94 | .173 | .96 | .467 | 0.23 | .632 |
| EDI-IA | .95 | .251 | .948 | .251 | 7.50 | .009 |
| DDF (TAS-20) | .95 | .321 | **.824** | **.001** | 2.16 | .148 |
| DIF (TAS-20) | .96 | .388 | **.864** | **.003** | **7.26** | **.010** |
| EOT (TAS-20) | .97 | .760 | .947 | .217 | 1.41 | .242 |
| Total score (TAS-20) | .97 | .655 | **.89** | **.011** | 0.84 | .365 |
| STAI-State | .94 | .193 | .97 | .633 | 3.80 | .058 |
| STAI-Trait | .95 | .225 | **.91** | **.024** | 1.45 | .235 |
| BDI-II | .95 | .380 | **.90** | **.021** | **27.5** | **<.001** |

***Note.*** Statistically significant differences between groups are shown in bold. BMI = Body Mass Index; RS = Restrained Scale; EDI-IA = Interoceptive Awareness subscale of the Eating Disorder Inventory; TAS-20 = Toronto Alexithymia Scale-20; DDF = difficulties describing feelings; DIF = difficulties identifying feelings; EOT = externally oriented thinking; STAI = Spielberger State Trait Anxiety Index; BDI-II = Beck Depression Inventory.

**Table S2.** Box’s *M* test and Mauchly’s test of sphericity for ANOVAs performed

| **Dependent variables** | **Box’s *M* test** | | | **Mauchly’s test** | |
| --- | --- | --- | --- | --- | --- |
|  | ***M*** | ***F*** | ***p*** | ***W*** | ***p*** |
| P300 Frontal | 28.47 | 1.17 | .269 | **.849** | **.023** |
| P300 Central | 27.98 | 1.15 | .289 | .95 | .341 |
| LPP Frontal | 31.10 | 1.28 | .178 | .96 | .358 |
| LPP Central | 27.98 | 1.15 | .289 | .95 | .341 |

***Note.*** Statistically significant differences between groups are shown in bold.

**Table S3.** *Correlations between the self-report and the electrocortical variables*

| **Variable** | | | **BMI** | | **BDI** | | **STAI-S** | | **STAI-T** | | **EDI-IA** | | **RS** | | **Effort SU** | | **Delta arousal** | | **TAS-20 (DDF)** | | **TAS-20 (DIF)** | | **TAS-20**  **(EOT)** | | **TAS-20 (Total)** | |  |
| --- | --- | --- | --- | --- | --- | --- | --- | --- | --- | --- | --- | --- | --- | --- | --- | --- | --- | --- | --- | --- | --- | --- | --- | --- | --- | --- | --- |
| P300 Frontal | View | Neutral | | -.17 | | -.18 | | **-.35*** | | -.27 | | .23 | | -.26 | | **.41**** | | .20 | | -.17 | | **-.34*** | | -.27 | | **-.31*** | |
|  |  | Negative | | -.07 | | -.16 | | **-.38**** | | -.23 | | .18 | | -.20 | | **.31*** | | .21 | | -.17 | | -.27 | | **-.36*** | | **-.29*** | |
|  |  | Positive | | -.06 | | -.08 | | -.27 | | -.17 | | .10 | | -.14 | | **.32*** | | .23 | | -.12 | | -.18 | | -.23 | | -.19 | |
|  | Suppression | Neutral | | .09 | | **-.46**** | | **-.57**** | | **-.50**** | | **.50**** | | **-.49**** | | **.33*** | | .02 | | **-.45**** | | **-.53**** | | **-.58**** | | **-.58**** | |
|  |  | Negative | | -.21 | | -.16 | | **-.42**** | | -.23 | | .17 | | -.19 | | .07 | | .04 | | -.11 | | **-.30*** | | **-.31*** | | -.27 | |
|  |  | Positive | | **-.48**** | | -.15 | | **-.44**** | | -.23 | | .16 | | -.20 | | .14 | | -.01 | | -.02 | | **-.32*** | | -.22 | | -.24 | |
| LPP Frontal | View | Neutral | | **-.42**** | | -.25 | | **-.40**** | | **-.35*** | | **.37**** | | **-.38**** | | -.04 | | **-.36*** | | -.12 | | **-.49**** | | -.05 | | **-.32*** | |
|  |  | Negative | | **-.45**** | | -.20 | | **-.45**** | | -.28 | | .28 | | **-.30*** | | -.10 | | -.27 | | -.06 | | **-.43**** | | -.11 | | -.28 | |
|  |  | Positive | | **-.43**** | | -.15 | | **-.30*** | | -.25 | | .26 | | -.27 | | -.18 | | **-.43**** | | -.01 | | **-.37*** | | .09 | | -.18 | |
|  | Suppression | Neutral | | -.24 | | **-.37*** | | **-.50**** | | **-.43**** | | **.48**** | | **-.45**** | | -.14 | | **-.45**** | | -.23 | | **-.55**** | | -.22 | | **-.43**** | |
|  |  | Negative | | **-.41**** | | -.27 | | **-.47**** | | **-.35*** | | **.36*** | | **-.35*** | | -.21 | | **-.41**** | | -.11 | | **-.48**** | | -.10 | | **-.32*** | |
|  |  | Positive | | **-.61**** | | -.17 | | **-.39**** | | -.24 | | .24 | | -.26 | | -.19 | | **-.38**** | | .02 | | **-.39**** | | .01 | | -.20 | |
| P300 Central | View | Neutral | | -.23 | | **-.33*** | | **-.46**** | | **-.41**** | | **.36*** | | **-.38**** | | **.46**** | | .21 | | -.26 | | **-.48**** | | **-.42**** | | **-.45**** | |
|  |  | Negative | | **-.38**** | | **-.32*** | | **-.48**** | | **-.34*** | | **.32*** | | **-.35*** | | **.34*** | | .18 | | -.24 | | **-.48**** | | **-.47**** | | **-.45**** | |
|  |  | Positive | | **-.51**** | | **-.34*** | | **-.47**** | | **-.35*** | | **.34*** | | **-.39**** | | **.36*** | | .18 | | -.24 | | **-.50**** | | **-.45**** | | **-.46**** | |
|  | Suppression | Neutral | | -.07 | | **-.42**** | | **-.50**** | | **-.43**** | | **.38**** | | **-.40**** | | **.47**** | | .28 | | **-.40**** | | **-.44**** | | **-.64**** | | **-.52**** | |
|  |  | Negative | | **-.44**** | | **-.39**** | | **-.59**** | | **-.41**** | | **.35*** | | **-.40**** | | .28 | | .23 | | **-.30*** | | **-.51**** | | **-.47**** | | **-.49**** | |
|  |  | Positive | | **-.57**** | | -.24 | | **-.51**** | | **-.30*** | | .19 | | -.27 | | **.32*** | | .20 | | -.10 | | **-.36*** | | **-.33*** | | **-.31*** | |
| LPP Central | View | Neutral | | **-.55**** | | **-.45**** | | **-.59**** | | **-.52**** | | **.50**** | | **-.52**** | | .05 | | **-.29*** | | -.24 | | **-.62**** | | -.26 | | **-.48**** | |
|  |  | Negative | | **-.64**** | | **-.39**** | | **-.56**** | | **-.42**** | | **.40**** | | **-.43**** | | .05 | | -.16 | | -.18 | | **-.56**** | | **-.31*** | | **-.44**** | |
|  |  | Positive | | **-.67**** | | **-.36*** | | **-.50**** | | **-.40**** | | **.38**** | | **-.42**** | | .05 | | -.19 | | -.15 | | **-.53**** | | -.23 | | **-.40**** | |
|  | Suppression | Neutral | | **-.40**** | | **-.44**** | | **-.58**** | | **-.47**** | | **.48**** | | **-.48**** | | -.05 | | **-.34*** | | -.25 | | **-.57**** | | **-.35*** | | **-.47**** | |
|  |  | Negative | | **-.50**** | | **-.53**** | | **-.69**** | | **-.56**** | | **.53**** | | **-.56**** | | .01 | | -.23 | | **-.32*** | | **-.65**** | | **-.35*** | | **-.54**** | |
|  |  | Positive | | **-.70**** | | -.25 | | **-.51**** | | **-.31*** | | .22 | | **-.30*** | | -.12 | | -.26 | | .00 | | **-.37*** | | -.08 | | -.22 | |

*Note.* BMI = body mass index; RS = Restrained Scale; EDI-IA = Interoceptive Awareness subscale of the Eating Disorder Inventory; TAS-20 = Toronto Alexithymia Scale-20; DDF = difficulties describing feelings; DIF = difficulties identifying feelings; EOT = externally oriented thinking; STAI = Spielberger State Trait Anxiety Index; BDI-II = Beck Depression Inventory. * indicates *p* < .05. ** indicates *p* < .01. Statistically significant relationships are shown in bold.
